# Comment on Wang et al.: Unaddressed Multiple Comparisons Risk Inflating Type, Error in Cardiovascular Specialty Nursing Research

**DOI:** 10.1002/clc.70409

**Published:** 2026-07-04

**Authors:** Awais Mehmood, Abdulrehman Bajwa

**Affiliations:** ^1^ Continental Medical College Lahore Pakistan; ^2^ University of Health Sciences Lahore Pakistan

To the Editor,

We read with interest the recent article by Wang et al. in Clinical Cardiology, examining humanistic concept‐based cardiovascular specialty care across medication adherence, glycolipid metabolism, psychological status, self‐management, and quality of life [1]. The multidimensional evaluation of patient‐centered nursing outcomes is a genuine strength, and the clinical question is timely.

We do, however, have a concern about the multiple testing strategy. The authors simultaneously tested 16 or more outcomes: six glycolipid parameters, two anxiety subscales, four ESCA domains, and four GQOLI‐74 domains each against *α* = 0.05, with no correction applied. When conducting 16 independent tests at this threshold, the familywise probability of at least one false positive exceeds 56% (1−[0.95]^16^). Several reported *p* values including medication adherence (*p* = 0.026), FPG (*p* = 0.013), TC (*p* = 0.005), and LDL‐C (*p* = 0.009) would not survive Bonferroni correction (adjusted threshold: *p* < 0.003). Even the more lenient Benjamini−Hochberg false discovery rate procedure would likely render some of these nonsignificant. The authors acknowledge this in their discussion, but acknowledgment without adjustment leaves the findings materially vulnerable to overinterpretation.

This issue is well‐documented. Bender and Lange [2] have outlined practical frameworks for multiplicity adjustment in clinical studies, and Bland and Altman [3] have long cautioned against applying simultaneous significance thresholds across correlated endpoints without accounting for inflation.

We suggest that future work pre‐specify one or two primary outcomes for confirmatory analysis, apply false discovery rate correction to secondary comparisons, and report effect sizes with 95% confidence intervals alongside *p* values. These steps would considerably strengthen what is otherwise a well‐conceived and clinically relevant contribution.

## Data Availability

The data that support the findings of this study are available in the Supporting Material of this article.
